# A Repeated Measures Experiment of School Playing Environment to Increase Physical Activity and Enhance Self-Esteem in UK School Children

**DOI:** 10.1371/journal.pone.0108701

**Published:** 2014-09-29

**Authors:** Carly Wood, Valerie Gladwell, Jo Barton

**Affiliations:** 1 School of Biological Sciences, University of Essex, Colchester, United Kingdom; Maastricht University Medical Centre, The Netherlands

## Abstract

School playtime provides daily opportunities for children to be active outdoors, but only makes small contributions to physical activity (PA) requirements. Natural environments facilitate unstructured PA and children report a preference for play in nature. Thus, play on the school field might encourage children to be more active during playtime. The primary aim of this study was to examine the impact of the school playing environment on children's PA. Descriptive data and fitness were assessed in 25 children aged 8–9 years from a single primary school. Over two consecutive weeks participants were allocated to either play on the school field or playground during playtime. The order of play in the two areas was randomised and counterbalanced. Moderate to vigorous PA (MVPA) was assessed during playtime on the last two days of each week using accelerometers. There was a significant interaction of environment and sex on MVPA during morning play (F(1,22) = 6.27; P<0.05; n_p_^2^ = 0.222), but not during lunch (P>0.05; n_p_^2^ = 0.060) or all of playtime combined (P>0.05; n_p_^2^ = 0.140). During morning play boys were significantly more active than girls on the playground (t(23) = 1.32; P<0.01; n^2^ = 0.291), but not on the field (P>0.05; n^2^ = 0.071). For lunch (F(1,22) = 24,11; P<0.001; n_p_^2^ = 0.523) and all of playtime combined (F(1,22) = 33.67; P<0.001; n_p_^2^ = 0.616) there was a significant effect of environment. There was also a significant main effect of sex during lunch (F(1,22) = 11.56; P<0.01; n_p_^2^ = 0.344) and all of playtime combined (F(1,22) = 12.37; P<0.01; n_p_^2^ = 0.371). MVPA was higher on the field and boys were more active than girls. Play on the field leads to increases in MVPA, particularly in girls. The promising trend for the effect of the natural environment on MVPA indicates that interventions aimed at increasing MVPA should use the natural environment and that schools should encourage greater use of their natural areas to increase PA.

## Introduction

The health benefits of engaging in physical activity (PA) during childhood include enhanced fitness, cognitive function and bone health; reduced body fatness, motor skill development, and favourable cardiovascular and metabolic disease risk profiles [1]–[4]. Being active during childhood can also improve self-esteem and reduce symptoms of anxiety and depression [5]–[7]. Participation in PA in youth is of great importance as PA may track into adulthood where adequate levels of PA are protective against many chronic diseases [8]. However, in the UK approximately 75% of boys and 80% of girls aged 5–10 years are not meeting the daily recommendation of 60 minutes of moderate to vigorous physical activity (MVPA) [9].

Unstructured play is also an essential part of childhood which enables children to develop a relationship with their surrounding environment and enhances social skills, co-ordination and strength [10]. Outdoor environments facilitate play and are associated with increased levels of PA [11], [12]. Thus, children should be provided with daily opportunities to play outdoors. The school environment provides such an opportunity through the provision of playtime. Playtime normally takes place on the concrete school playground and lasts for at least one hour per day [12]. However, universally playtime is reported to make relatively small contributions to children's overall daily activity requirements [13]. In the UK, only one known study has reported the contribution of playtime to overall activity requirements, with contributions being as low as 4.5% [14].

A number of studies have successfully increased playtime PA through the introduction of interventions such as sports or games equipment [15], playground markings [16], [17], fitness breaks [18], [19] and playground structures [20]. However, these types of interventions tend to facilitate structured rather than unstructured PA [21]. Unstructured PA is essential to childhood development [10] and therefore needs to be encouraged during playtime.

Natural environments can encourage unstructured play and may therefore play a role in facilitating unstructured PA during playtime [21]. Natural environments provide large open spaces which encourage individuals to be active, whilst areas lacking nature may restrict PA due to limited space and parental fears over crime and road traffic [22]–[24]. Children report a preference for play in natural environments [10], with nature facilitating more imaginative and inventive play [10], [22], [25]. Furthermore, adolescents living in urban settings with access to green spaces such as parks are more likely to be physically active than their peers without park access [26], indicating that all forms of nature can be used as a tool for engaging youth in PA. Thus, if school playtime were performed on the school field it is possible that children's PA levels would be increased. To date, there is a lack of data quantifying the impact of natural environments on levels of PA in children, particularly within the school setting.

Performing PA in a natural environment (“Green Exercise”) has also been demonstrated to provide improvements in self-esteem in adults [27]–[29], whether participants are simply viewing scenes of nature or directly interacting with natural environments. Studies in adolescents and children suggest that Green Exercise has no such additive effect on self-esteem compared to exercise in other environments [30]–[32]. However, the only known study in children examined the impact of a green playtime intervention consisting of orienteering [32]. The task orientated, structured nature of orienteering may not facilitate the green exercise effect [33]. Unstructured free play in a natural environment may allow greater interaction with the environment, thus benefiting self-esteem. Low self-esteem is a common occurrence in many forms of mental illness [34], thus methods of improving self-esteem in children are important for mental health.

The primary aim of this study was to examine the impact of the school playing environment on children's PA using a counterbalanced, randomised cross-over design. The secondary aim was to determine whether the playing environment influenced short term changes in self-esteem.

## Methodology

### Ethics Statement

The University faculty ethical review committee approved the study. Participants from one urban primary school were recruited to take part. The school holds 575 children aged 3 to 11 years and has extensive outdoor areas, including three playgrounds, a garden area, an environmental area, an outdoor classroom and large grassed field. The school was selected for this study due to its large natural and built areas, and their comparative sizes. Twenty five children from one class volunteered, including 12 boys (mean±SD) aged 8.7±0.3 years and thirteen girls (mean±SD) aged 8.5±0.2 years. All participants were deemed fit and healthy by the school and individual assent and parental consent was obtained.

### Experimental Procedures

Initially participants' basic anthropometric data were collected comprising stature to the nearest 0.1 cm, with the participant barefoot and mass to the nearest 0.1 kg. Body Mass Index (BMI) and BMI z-scores relative to the individual's age and sex were also calculated [35]. Participants also completed a version of the FITNESSGRAM pacer test, which is a valid method by which to assess aerobic fitness in this age group [36].

Over two consecutive weeks participants were allocated to either play on the school field or the playground during morning and lunch playtime. Participants were first grouped according to whom they would normally play with during playtime. The order in which these groups played in the two areas was then randomised and counterbalanced to eliminate any order effects, with approximately half of the group playing in each area at any one time. In order to randomise playing groups to the two playing areas a representative from each group selected a piece of paper from a bag, numbered with either 1 or 2. Participants who selected number 1 were allocated to the field first, whilst participants who selected number 2 were allocated to the playground first. The school field was surrounded by trees and bushes, whilst the playground consisted of concrete areas surrounded by school buildings. Participants could not see the playground from the field; however the boundary of the field was visible from some areas of the playground. Participants were instructed to play as normal and were free to engage in their chosen activities. Morning playtime lasted for 15 minutes, whilst lunch playtime lasted for one hour including the time taken to eat lunch (approximately 30 minutes).

### Measurement of Physical Activity

PA was monitored during morning and lunch playtime on the final two days of each week using Actigraph GT1M accelerometers. Accelerometers were placed on the right hip and set to record at a 1 second EPOCH. Data was downloaded using the Actilife programme (V4.4.1) and data reduction and transformation took place via Actisci (V0.99b5). Accelerometer cut points developed by Trueth et al [37], with the Corder adjustment [38] were applied to the raw accelerometer counts in order to determine the amount of time spent in MVPA.

### Assessment of Self-esteem

Self-esteem was assessed prior to the start of the project and after lunch time on the final monitoring day of each week using the one-page 10-item Rosenberg self-esteem scale [39]. The scale was slightly modified to ensure the language could be understood by the age-group involved. Participants are normally asked how they feel about themselves and whether they strongly agree, agree, disagree or strongly disagree with a list of 10 statements. This was amended to very true, true, not true or definitely not true. Some statements were also modified to make the language more comprehensible, for example “I am able to do things as well as most other people” was changed to “I can do things as well as most other children” [33]. Participants were asked to complete the questionnaire independently and honestly.

### Statistical Analysis

Independent t-tests were used to compare anthropometric measures in boys and girls. Mixed ANOVA examined the effect of the environment and sex on: MVPA during morning playtime, lunch playtime and all of playtime combined; and also on the change in participants' self-esteem scores. Significance was set at a p value of 0.05 throughout the analysis (see Raw data S1 for SPSS raw data sheet). Effect sizes for ANOVA outcomes are presented as partial eta-squared (n_p_^2^) and for t-tests as eta-squared (n^2^).

## Results

All measured parameters in boys and girls were not significantly different, except for stature where boys were significantly taller (t(23) = 2.34; P<0.05; n^2^ = 0.193) (Table 1). The BMI z-scores indicated that both boys and girls had a slightly above average BMI for their age and sex, whilst the 20mSRT z-score indicated that boys' fitness was slightly above average for their age and sex and girls was slightly below average.

**Table 1:** Descriptive anthropometric and fitness data for the sample.

Measure	Boys (n = 12)	Girls (n = 13)	All (n = 25)
Age (yrs)	8.7±0.3	8.5±0.2	8.6±0.3
Stature (m)	1.37±0.06*	1.32±0.03	1.34±0.05
Mass (kg)	32.9±5.4	30.2±2.4	31.5±4.3
BMI (kg.m^−2^)	17.6±1.8	17.3±1.2	17.4±1.5
BMI (z-score)	0.77±0.79	0.48±0.52	0.62±0.67
20mSRT Shuttles (No.)	25.2±12.0	18.9±5.9	21.9±9.7
20mSRT Speed (Km.h^−1^)	9.8±0.6	9.5±0.3	9.7±0.5
20mSRT (Z- score)	−0.14±0.65	0.10±0.39	−0.02±0.53

*indicates a significant sex difference in stature (P<0.05).

BMI = body mass index, 20mSRT = twenty metre shuttle run test.

For morning playtime mixed ANOVA revealed a significant interaction effect for MVPA due to the environment and sex (F(1,22) = 6.27; P<0.05; n_p_^2^ = 0.222). Post-hoc independent t-tests revealed that boys were significantly more active than girls on the playground (t(23) = 1.32; P<0.01; n^2^ = 0.291), but not on the field (P>0.05; n^2^ = 0.071). Both boys and girls increased their MVPA on the field compared to playground and MVPA was higher in boys than girls in both environments (Table 2). For lunch playtime mixed ANOVA revealed no significant interaction effect for MVPA due to the environment and sex (P>0.05; n_p_^2^ = 0.060), however there was a significant main effect for environment (F(1,22) = 24.11; P<0.001; n_p_^2^ = 0.523) and sex (F(1,22) = 11.56; P<0.01; n_p_^2^ = 0.344). MVPA increased in both boys and girls in the natural environment, with boys having higher MVPA than girls throughout (Table 2).

**Table 2:** Time (mins) spent in MVPA during playtime on the playground and field.

		Boys	Girls	All
**Morning playtime**	**Playground**	4.7±1.6 (3.8–5.5)	2.9±1.3* (2.1–3.8)	3.8±1.7 (3.2–4.4)
	**Field**	5.0±0.8 (4.6–5.4)	4.6±0.6 (4.2–5.0)	4.8±0.7# (4.5–5.1)
	**All**	4.8±1.0 (4.3–5.4)	3.8±0.9+ (3.2–4.3)	4.2±1.0 (3.8–4.7)
**Lunch playtime**	**Playground**	10.9±2.7 (9.4–12.4)	6.7±2.1 (5.3–8.1)	8.6±3.2 (7.7–9.8)
	**Field**	13.8±3.1 (11.6–16.0)	11.5±3.9 (9.5–13.5)	12.6±3.7# (11.1–14.1)
	**All**	12.3±2.1 (10.9–13.8)	9.1±2.5+ (7.7–10.4)	10.7±2.9 (9.4–11.9)
**All Playtime**	**Playground**	15.4±3.3 (13.5–17.3)	9.6±2.8 (7.7–11.4)	12.4±4.2 (11.1–13.8)
	**Field**	18.8±3.6 (16.3–21.2)	16.1±4.1 (13.8–18.5)	17.4±4.1# (15.7–19.2)
	**All**	17.1±2.7 (15.3–18.9)	12.9±3.0+ (11.1–14.6)	14.9±3.5 (13.3–16.4)

*indicates a significant difference between males and females MVPA on the playground during morning play (P<0.01).

# indicates a significant environmental effect (P<0.01 for morning playtime; P<0.001 for lunch and all playtime);

+ indicates a significant sex effect (P<0.01).

For all of playtime combined there was no significant interaction effect for MVPA due to environment and sex (P>0.05 n_p_^2^ = 0.140). There was a significant main effect for environment (F(1,21) = 33.67; P<0.001; n_p_^2^ = 0.616) and sex (F(1,21) = 12.37: P<0.01 n_p_^2^ = 0.371) (Table 2). Similarly to at lunch playtime; MVPA increased in the natural environment and was higher in boys than in girls.

The change in self-esteem from the pre- self-esteem score (Boys = 27.8±4.0; Girls = 29.1±6.0) was calculated for both the playground and field environments. Mixed ANOVA revealed no significant interaction effect for environment and sex (P>0.05; n_p_^2^ = 0.000). There was also no significant effect for the change in self-esteem due to the environment (P>0.05; n_p_^2^ = 0.000) or sex (P>0.05; n_p_^2^ = 0.000). The change in self-esteem following play on both the playground and field were similar in both boys and girls (Table 3).

**Table 3:** Change in self-esteem after play on the playground and field.

	Playground	Field	Total
**Boys**	0.50±2.72 (−1.73–2.73)	0.50±3.74 (−1.70–2.70)	0.50±2.80 (−1.20–2.20)
**Girls**	0.83±3.83 (1.20–2.87)	0.75±2.96 (−1.26–2.76)	0.79±2.38 (−0.76–2.34)
**All**	0.68±3.30 (−0.84–2.18)	0.64±3.26 (−0.87–2.12)	0.66±2.52 (−0.46–1.78)

## Discussion

The primary aim of this study was to examine the impact of the school playing environment on children's MVPA. Despite the fact that natural environments facilitate unstructured PA [21] and that children have a preference for play in nature [10]; the impact of the playing environment on PA in children has not been established.

The findings of our single school repeated measures experiment indicated that simply altering the playtime environment could result in an increase in MVPA. In fact, 61.6% of the variance in MVPA was due to the playing environment (indicating a large effect size) and participants engaged in 40% more MVPA when playing on the field compared to the playground, with play on the playground and field contributing 20% and 29% towards the daily activity requirement respectively. School playtime provides one of the most substantial opportunities for children to be active on a daily basis [12], yet research including this study suggests that it is largely underutilized [13], and that more PA could be accumulated during school playtimes. It is suggested that 40% of playtime should be spent in MVPA [12]. If we take into account the time taken to eat lunch (approximately 30 minutes) participants were provided with approximately 45 minutes of daily playtime. On the playground 28% of playtime was spent in MVPA compared to 39% on the field, resulting in an 11% increase. Some structured playtime interventions have resulted in increases in MVPA as low as 4.5% [20]. The results of this study therefore indicate that by simply altering the play environment school playtime can be more effectively used for its intended purpose. However, this study was conducted in a single school. Replication of the research in schools throughout the UK would be required to further explore this idea. Furthermore, if playtime interventions were to take place in a natural environment it is possible that playtime could make an even greater contribution to the daily activity requirement.

In addition to increasing the time spent in MVPA; play in the natural environment also closed the gap between the differences in boys and girls activity levels. It is widely acknowledged that boys engage in more PA than girls [9], and this pattern was evident on the playground and field during all playtime periods. However during play on the field boys only engaged in an extra 0.4 minutes, 2.3 minutes and 2.7 minutes of MVPA during morning, lunch and all of playtime respectively, compared to 1.8, 4.2 and 5.8 minutes on the playground respectively. Whilst the interaction effect between the environment and sex was only significant during morning playtime; there was a trend for higher increases in girls MVPA when playing on the field. Girls engaged in an extra 59% MVPA during morning play, 72% during lunch play and 68% for all of playtime combined when playing in nature. Thus for girls, playtime interventions which take place in natural environments are likely to be the most effective at increasing MVPA. However, participants were not given an option as to which environment they wanted to play in. It would be interesting for future research to establish children's environmental preferences and whether preferred environments are associated with increased MVPA.

### Secondary Aim

The secondary aim of this study was to determine whether playing in a natural environment provided additive benefits for children's self-esteem. The benefits of Green Exercise for self-esteem are widely acknowledged in adults [25], [29]; however there are no additive benefits for adolescent's self-esteem [30], [31], [33]. Whilst PA alone improves self-esteem in children [6]; the results of this study indicate that PA in natural environments does not have an additive effect. These findings support those of Barton et al [32], who found no additive benefit for self-esteem of a nature-based playtime intervention. This lack of effect can be attributed to children's low levels of interaction with nature. The current generation of youth spend less time interacting with nature than previous generations [10]; with only 10% of children having regular contact with nature compared to the 40% of adults who did so 30–40 years ago [41]. Young people spend more of their time indoors and may have developed a disconnection and lack of understanding as a consequence [10]. For benefits to occur it is proposed that an individual needs to be “connected” to nature in some way [42]. Thus children, like adolescents, might not have received additive benefits from the green exercise as they do not experience the same level of connection to the natural world as adults. This idea is only speculative; but would go some way to explaining the lack of additive benefits for self-esteem in both children and adolescents [30], [31]. However, it is possible that children need more direct interaction with nature to receive benefits. Simply playing within a natural environment is unlikely to promote direct engagement.

In a previous school-based Green Exercise study in adolescents it was also proposed that the school environment may not provide a suitable condition for participants to receive benefits from green exercise [31]. Self-esteem is suggested to be enhanced through the ability of natural spaces to provide a distraction from daily stresses [27], [31]. The school environment may actually provide a direct source of stress for children, thus performing green exercise within school may not have the same impact on self-esteem as performing green exercise outside of the school day.

### Limitations and Recommendations

The current study has a number of limitations. The Rosenberg Self-esteem scale is open to what is known as a ceiling and floor effect. Participants may rate themselves as having a high self-esteem at the start of the study, but feel better following the PA. As a high score has already been achieved; it may be difficult to quantify this improvement. In the current study the starting self-esteem score was 28.8, out of a possible 40. Participants were therefore experiencing a relatively high initial self-esteem, limiting the possible magnitude of improvements. Rosenberg's self-esteem scale is also typically used as a trait scale and may have reduced the studies sensitivity. Future research would benefit from the use of a state self-esteem scale [31], [43]. The novelty effect of wearing the accelerometers also needs to be considered, however the playing conditions were randomised in order to eliminate this effect. Furthermore, activity was measured at the end of each playing week to address the novelty of the change in playing area. The process of randomisation might have also introduced a degree of bias into the study as the participants were grouped according to whom they would normally play with prior to randomisation. However, if this had not been part of the randomisation process, the inability to play with normal friendship groups may have adversely affected MVPA and impacted on the ability of the study to assess the effect of the change in playing area on MVPA.

The use of one primary school could also be considered as a study limitation. It would be beneficial for future research to examine the impact of Green Exercise in schools from contrasting areas of the UK including urban, rural and sub-urban as research indicates that activity levels may vary between children from different areas [44]. Interventions requiring direct interaction with and use of nature would also be beneficial, as they may result in children developing a relationship with nature, which could in turn result in enhanced self-esteem.

### Conclusions

The current study assessed the impact of the playing environment on the PA and self-esteem of children from one primary school. The natural environment led to increases in MVPA during all playtime periods, and particularly in girls. However, the effects of green exercise on children's self-esteem were no greater when playing on the field. The promising trend for the effect of the natural environment on MVPA indicates that interventions aimed at increasing MVPA in line with recommendations should incorporate the use of natural environments. We therefore suggest that schools should encourage greater use of the school field and natural areas of the school in order to promote greater participation in PA amongst their students.

## Supporting Information

Raw data S1 **SPSS raw data sheet.**
(SAV)
